# Discovery and mechanism of a highly selective, antifungal acetyl CoA synthetase inhibitor

**DOI:** 10.21203/rs.3.rs-5619443/v1

**Published:** 2025-01-01

**Authors:** Andrew J. Jezewski, Katy M. Alden, Drashti G. Daraji, Charles L. Lail, Jonah Propp, Michael E. Heene, Andrew J. Fuller, Lijun Liu, Kevin P. Battaile, Noelle S. Williams, Bart L. Staker, Scott Lovell, Timothy J. Hagen, Damian J. Krysan

**Affiliations:** 1 Department of Pediatrics, Carver College of Medicine, University of Iowa, Iowa City, IA 52242; 2 Department of Chemistry and Biochemistry, Northern Illinois University, DeKalb, IL 60115; 3 Protein Structure and X-ray Crystallography Laboratory, University of Kansas, Lawrence, KS 66047; 4 NYX, New York Structural Biology Center Upton, NY 11973; 5 Department of Biochemistry, UT Southwestern Medical Center, Dallas, TX 75390; 6 Center for Global Infectious Disease Research Seattle Children’s Research Institute, Seattle, WA 98109; 7 Seattle Structural Genomics Center for Infectious Disease (SSGCID), Seattle, Washington, 98109, USA; 8 Department of Molecular Physiology and Biophysics, Carver College of Medicine, University of Iowa, Iowa City, IA 52242.

## Abstract

Acetyl CoA synthetases (ACS) have emerged as drug targets for the treatment of cancer, metabolic diseases as well as fungal and parasitic infections. Although a variety of small molecule ACS inhibitors have been discovered, the systematic optimization of these molecules has been slowed by a lack of structural information regarding their mechanism of inhibition. Through a chemical genetic-based, synthetic lethal screen of the human fungal pathogen *Cryptococcus neoformans*, we identified an isoxazole-based ACS inhibitor with antifungal activity and exquisite selectivity for the *C. neoformans* Acs1 relative to human ACSS2 as well as other fungal ACSs. Xray crystallographic characterization of the isoxazole-*Cn*Acs1 complex revealed that the isoxazole functions as an acetyl CoA mimic and occupies both the acetyl- and CoA-binding sites of *Cn*Acs1. Consistent with this novel mode of inhibition, the isoxazoles display uncompetitive inhibition kinetics that are similar to antimalarial ACS inhibitors also proposed to target the CoA binding site. Consequently, these data provide structural and mechanistic insights into the remarkable selectivity of Acetyl CoA pocket-targeting ACS inhibitors. In addition, these data provide strong proof-of-principle that targeting fungal and parasitic ACSs for the development of novel anti-infectives can be achieved with high selectivity and, thereby, low host toxicity.

## Introduction

Each year fungal infections affect millions of people across the globe and cause diseases ranging from superficial skin dermatoses to life-threatening diseases of the bloodstream and deep organs such as liver, kidney and brain (1). The majority of people at-risk for invasive fungal infections have altered immune function due to other diseases such as HIV/AIDs or immunosuppressive drugs used to treat cancer, inflammatory diseases or manage organ transplantation (2). In addition, people with fully functional immune systems, however, are at risk for life-altering fungal infections such as fungal keratitis, a common cause of infection-related vision loss, or chronic aspergillosis-associated with asthma (1, 2). Finally, human fungal infections are caused by an evolutionarily divergent set of pathogens with a broad range of pathobiological characteristics and features. Together, these features of human fungal diseases make them challenging to treat.

Currently, only three classes of antifungal drugs are available as primary therapies for life-threatening fungal infections (3): 1) polyenes such as amphotericin B; 2) azoles including fluconazole and voriconazole; and 3) echinocandin 1,3-β-glucan synthase inhibitors exemplified by micafungin. Other drugs such as 5-flucytosine or the novel triterpenoid 1,3-β-glucan synthase inhibitor ibrexafungerp are used as adjuvant agents (5-flucytosine, ref. 4) or currently limited to the treatment of mucosal infections (ibrexafungerp, ref. 5). The number of mechanistically distinct classes of antifungal drugs is very limited compared to those available for treating bacterial infections. For example, there are more distinct classes of antibiotics with activity against methicillin-resistant *Staphylococcus aureus* (MRSA) than classes of antifungal drugs in total. As resistance to frontline antifungal drugs continues rise in species such as *Candida auris* and *Aspergillus fumigatus* (6), the urgency of developing chemically and mechanistically novel antifungal drugs increases.

The unmet clinical need for new antifungal drugs has not gone un-noticed and, happily, two mechanistically and structurally novel candidates are currently in Phase II/III clinical trials. Specifically, fosmanogepix is a broad-spectrum agent that targets GPI anchor biosynthesis critical for fungal cell wall biosynthesis (7) while olorofim primarily has activity against difficult-to-treat molds and selectively inhibits fungal dihydroorotate dehydrogenase (8). However, even if these agents advance to the clinic, the threat of resistance remains acute with only 4–5 drugs available to treat the broad variety of human fungal pathogens. Indeed, agricultural fungicides based on dihydroorotate dehydrogenase inhibition are already proceeding to use and could generate olorofim-resistant *A. fumigatus* before olorofim finishes Phase III clinical trials (9). Therefore, the discovery and development of new antifungal drug candidates remains an important endeavor for medical mycology.

Acetyl CoA synthetase (ACS) is an ANL-family, adenylating enzyme (10) that converts acetate to the key metabolic molecule, acetyl CoA (AcCoA). In mammalian cells, the majority of AcCoA is derived from glucose via citrate generated by the tricarboxylic acid (TCA) cycle (11). TCA-derived citrate is exported from the mitochondria and converted to AcCoA by ATP-citrate lyase (ACL) in the cytosol and the nucleus (12). Overall, acetate-derived AcCoA appears to represent ~10% of the total pool in human cells under a normal state of homeostasis while AcCoA derived from glucose/ACL makes up the majority of the cellular pool (11). The biochemical origin of AcCoA is reversed in multiple cancer types and acetate-derived AcCoA makes up the majority of the pool (13, 14). As such, the primary human ACS, ACSS2, has emerged as a cancer chemotherapy target and one ACSS2 inhibitor progressing to early-stage clinical trials (14). Importantly, ACSS2 is not essential in mammals (13) because ACL maintains the AcCoA pool under normal homeostasis; therefore, ACCS2 inhibitors have a reduced likelihood of causing toxicity in humans.

ACS has also generated interest as a target for the development of anti-infective agents including antifungal (15, 16) and anti-malarial agents (17, 18). Specifically, we found previously that the pyrazole AR-12 inhibits fungal ACS (15), has broad spectrum antifungal activity and is efficacious in combination with fluconazole a mouse model of disseminated cryptococcosis (16). These data provided validation of ACS as a potential antifungal drug target. Unfortunately, the pharmacology of AR-12 is not suitable for further pre-clinical development. Furthermore, while a significant portion of the antifungal activity of AR-12 is due to ACS inhibition, the molecule has other targets (19) that may contribute to its antifungal activity and, more importantly, to its mammalian cell toxicity.

ACS, however, remains an attractive antifungal drug target for the following reasons. First, medically important yeast such as *Candida albicans* and non-albicans *Candida* spp. lack ACL enzymes and, therefore, are dependent on ACS for the generation of critical pools of AcCoA (20). Multiple genetic analyses have provided strong support for the conclusion that ACS enzymes are essential in *C. albicans* and *C. glabrata* (21, 22) and the assertion that other *Candida* spp. such as *C. auris* will also require ACS for viability.

Second, fungal ACS enzymes appear to play important roles in carbon metabolism during mammalian infection in species of fungi that also express the ACL enzymes. For example, Hu et al. have shown that deletion of *CnACS1*, the only ACS expressed in the human fungal pathogen *C. neoformans* (23), reduces virulence in a mouse model of cryptococcosis (24). We have also demonstrated that *C. neoformans* strains lacking *CnACS1* are more susceptible to fluconazole during mouse infection (23), suggesting that *Cn*Acs1 inhibitors would be useful in combination therapy. Based on these considerations, we undertook a chemical-genetic, high throughput screening approach to identify novel fungal ACS inhibitors.

**Figure 1.**
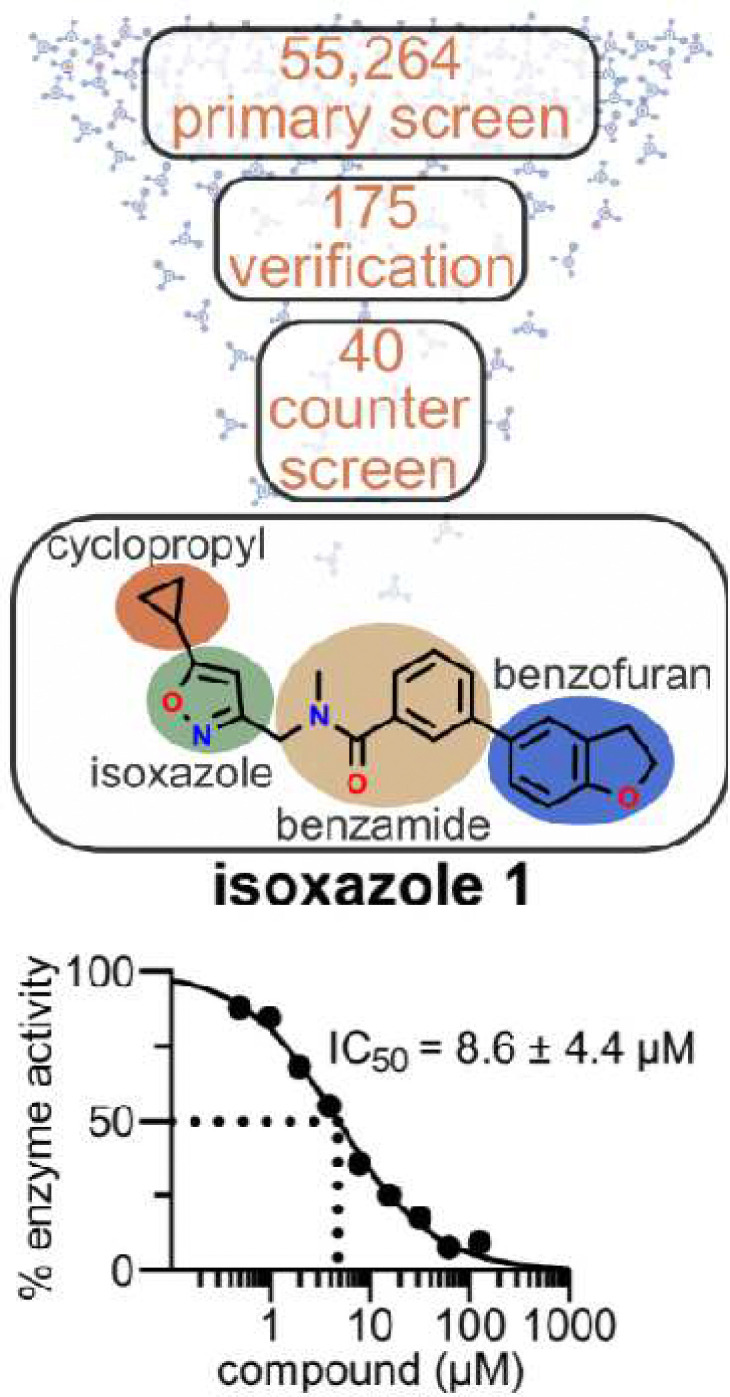
Outline of CnAcs1 inhibitor screen and validation strategy leading to identification of isoxazole 1. The number of compounds evaluated in the primary screen, hit validation, and counter screening steps of the screening campaign are shown. The structure of the hit isoxazole **1** is provided with the functionally distinct regions of the molecule highlighted. The initial IC_50_ of isoxazole **1** towards CnAcs1 is indicated; the curve is representative of two independent experiments with the IC_50_ and error shown.

In vitro, neither *CnACS1* nor *CnACL1* genes is essential but they appear to be synthetically lethal based on our inability to generate the corresponding *acs1*Δ *acl1*Δ double mutant (23). We took advantage of this synthetic lethal relationship to bias a cell-based, phenotypic screen toward potential *Cn*Acs1 inhibitors by screening an *acl1*Δ mutant against a library of small molecules (Fig. 1). As described below, this approach led to the identification of a structurally novel ACS inhibition (isoxazole **1**) with high selectivity for *Cn*Acs1 and in vitro antifungal activity. Xray crystallography and biochemical analysis indicate that **1** displays a unique mode of enzyme inhibition distinct from previously reported ACS inhibitors. These studies not only further validate ACS as an antifungal drug target but also highlight a surprisingly high level of target specificity that ACS inhibitors show toward fungal and human ACS enzymes despite the high structural and sequence conservation.

## Results

### Chemical-genetic, synthetic lethal screen in *C. neoformans* identifies a *Cn*Acs1 inhibitor.

As introduced above, we took advantage of the synthetic lethality of the *acs1*Δ *acl1*Δ mutant in *C. neoformans* (23) to design a whole-cell, high throughput screen to identify potential inhibitors of *Cn*Acs1 (Fig. 1). To do so, we employed a two-stage, screen-counter screening strategy. First, the *acl1Δ* mutant was screened against a compound library for molecules that inhibit growth in standard yeast peptone-2% dextrose (YPD) medium. The synthetic lethal relationship between *ACS1* and *ACL1* was expected to sensitize the *acl1*Δ mutant to *Cn*Acs1 inhibitor. Second, we counter-screened hits against WT cells in the same medium. The *acs1*Δ mutant has no growth phenotype in YPD and, therefore, on-target *Cn*Acs1 inhibitors should have reduced antifungal activity against strains expressing both *ACS1* and *ACL1*. Third, we also determined the antifungal activity of hits that advanced through the first two steps against WT strains in medium containing acetate as the sole carbon source. Under these conditions, *CnACS1* is essential for growth (23, 24). We expected that this screening funnel would skew hits toward on-target Acs1 inhibitors; we also hypothesized that the highly conserved nature of fungal ACS (25) enzymes would allow our strategy to identify broad-spectrum ACS inhibitors.

We optimized growth conditions for the *acl1*Δ mutant in 384-well format at 30°C in YPD for 48hr using cell density (OD_600_) as the readout for fungal growth. Fluconazole was used as positive control to validate the robustness of the assay for the detection of antifungal activity. Full plate assays with alternating rows of DMSO (1%) and fluconazole (64 μg/mL) generated a Z’ score of 0.65 which is compatible with high throughput screening (**Fig. S1**). A library of 55,264 drug-like DIVERSet purchased from ChemBridge was screened to yield 175 molecules (0.3% hit rate) that reduced growth of the *acl1*Δ mutant (Z ≥ 3) relative to the mean of each plate (Fig 1). This set of hits was re-tested under the screening conditions to identify molecules that inhibited growth by 50% in the primary screen and in the validation screen (Fig. 1).

**Figure 2.**
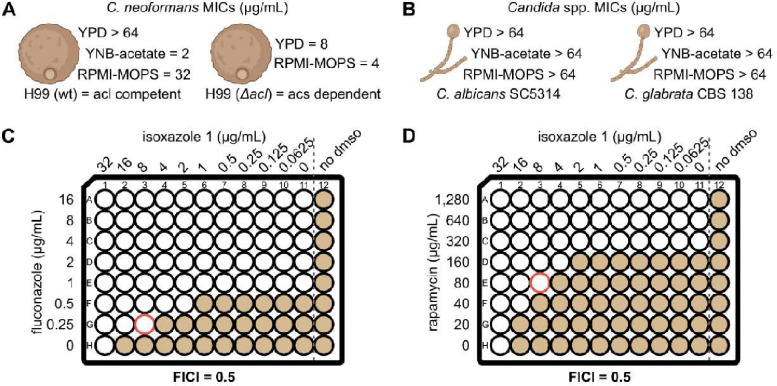
Antifungal activity of isoxazole 1. **A.** The minimum inhibitory concentration (MIC) of **1** against *C. neoformans* reference strain H99 and *acl1Δ* mutant strains in YPD, YNB+2%acetate, and RPMI-MOPS buffer at 37°C. The values were identical for three independent experiments performed in technical duplicate. **B.** MIC values against *C. albicans* reference strain SC5314 and *C. glabrata* CBS138. Fractional inhibitor concentrations against H99 for fluconazole (**C**) and rapamycin (**D**). Growth in wells is indicated by tan fill while empty wells indicate no growth. The wells with red outline indicate the fractional inhibitory concentration (FIC). Fractional inhibitor concentration index (FICI) ≤ 0.5 indicates synergy.

Independent samples of the 40 molecules that met these criteria were re-purchased and counter-screened against WT (strain H99) and the *acl1*Δ mutant under screening conditions. Five molecules showed decreased antifungal activity against the WT strain compared to the *acl1*Δ mutant. Finally, the antifungal activity of these five molecules against the WT strain was determined in minimal medium with 2% acetate as the carbon source. Of these five, isoxazole **1** was dramatically more active in acetate medium (Minimum Inhibitory Concentration (MIC), 2 μg/mL) relative to rich medium (MIC >64 μg/mL) (Fig. 2A). To further confirm the structure and reproducibility of isoxazole **1**, it was re-synthesized using the route described in **Fig. S2** and its activity confirmed using growth assays with WT, the *acl1*Δ mutant, and acetate-containing media. Finally, isoxazole **1** inhibited *Cn*Acs1 enzyme with an IC_50_ of 8.6 ± 4.4 μM using our previously reported kinetic assay of *Cn*Acs1 enzyme activity (Fig. 1, **S3**, ref. 25). These data strongly support the conclusion that isoxazole **1** inhibits *Cn*Acs1.

### Isoxazoles 1 & 2 have anticryptococcal activity in host-like media and are synergistic with fluconazole in vitro.

Although isoxazole **1** did not show antifungal activity against WT *C. neoformans* in rich medium, we re-tested it using standard clinical microbiology (CLSI) antifungal susceptibility testing conditions (RPMI medium buffered with 0.165M MOPS, ref. 26). Under these conditions, **1** inhibited the growth of H99 with a MIC of 32 μg/mL (Fig. 2A). RPMI is tissue culture medium and much more “host-like” than rich YPD broth (27). The increased activity of **1** in RPMI relative to rich YPD medium is likely due to the difference in nutrient availability. Supporting that hypothesis, we previously showed that host-like in vitro conditions induce *CnACS1* expression (23), suggesting that the Acs1-derived pool of AcCoA may become more important under these conditions. The *acs1*Δ mutant does not show strong growth defects under these conditions, suggesting that rapid loss of Acs1 function by chemical inhibition cannot be compensated by the cell.

Surprisingly, **1** did not have antifungal activity against *C. albicans* or *C. glabrata*, two *Candida* spp. which lack ACL and, therefore, would be predicted to be more susceptible to ACS inhibition (Fig. 2B). The primary sequences of ACS enzymes are highly conserved across multiple pathogenic fungi including *C. neoformans*, *C. albicans* and *C. glabrata* (**Fig. S4**). Additionally, crystallography of the *C. neoformans* and *C. albicans* proteins also show that the secondary and tertiary protein structures of the enzymes are also very similar to one another as well as to ACS enzymes from other species (25). These observations indicate that **1** has unexpectedly high selectivity for *Cn*Acs1 or that it does not penetrate well into the *Candida* spp.

As we previously reported (23), the *acs1*Δ mutant shows modestly increased susceptibility to fluconazole (2-fold) relative to WT in vitro. In a mouse model of cryptococcosis, the effect of fluconazole on the *acs1*Δ mutant is 0.5 log_10_ greater than WT. Consistent with these genetic data, isoxazole **1** is synergistic with fluconazole in vitro with a FICI of 0.5 (Fig. 2C). In contrast, **1** is indifferent in its interactions with amphotericin B (FICI = 2) and 5-flucytosine (FICI = 2), the other two antifungal drugs used to treat CM (28). In mammalian systems, Acs1 activity is regulated by the Target-of-Rapamycin (TOR) pathway (29). Consistent with these previous findings, **1** is also synergistic with rapamycin against *C. neoformans* (FICI = 0.5, Fig. 2D).

**Figure 3.**
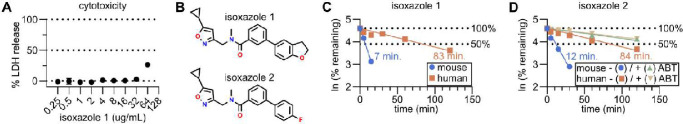
In vitro cytotoxicity and microsome stability of isoxazoles. **A.** HepG2 cells were exposed to the indicated concentrations of isoxazole **1** for 24hr. The release of lactate dehydrogenase (LDH) into the medium was determined as described in materials and methods and normalized to detergent-induced lysis (100% lysis). Data are means of two independent experiments performed in technical triplicate with error bars indicating standard deviation. **B.** Structures of isoxazole **1** and **2**. **C.** In vitro stability of isoxazole **1** in liver and mouse microsomes. The time indicate t_1/2_ in minutes. **D.** In vitro stability of isoxazole **2** in the presence and absence of pan-cytochrome inhibitor 1-amino-benzotriazole (ABT).

There has been substantial interest in identifying molecules that synergize with fluconazole for the treatment of *C. neoformans* (28). Based on our previously reported genetic data indicating that genetic disruption of Acs1 increases fluconazole efficacy and synergy of isoxazole **1** with fluconazole (23), we further investigated the in vitro toxicity and in vitro pharmacodynamic properties of **1** as a prelude to potential efficacy studies in a mouse model of infection. As shown in Fig. 3A, **1** shows very little to no toxicity up to the limit of its solubility against Hep2G cells using an LDH assay. Inspection of **1** reveals a potential metabolic liability in the form of an oxidation-prone, electron-rich benzofuran ring. Therefore, we synthesized isoxazole **2** in which the benzofuran was removed and a fluorine-substituent was placed at the *para*-position of the aryl ring (Fig. 3B). The antifungal activity of **2** (MIC 32 μg/mL, RPMI) was maintained.

Consistent with our concerns, isoxazole **1** was rapidly metabolized by murine microsomes (T_1/2_ 7 min) (Fig. 2C). Unfortunately, the t_1/2_ of the isoxazole **2** was only slightly longer (t_1/2_ 12 min, Fig. 2D). This metabolism was blocked by the addition of the pan-cytochrome P450 inhibitor 1-amino-benzotriazole (t_1/2_ >120 min, ref. 30), confirming the susceptibility of **2** to cytochrome-mediated metabolism (Fig. 2D). Both **1** and **2** showed longer t_1/2_ in human microsomes (t_1/2_ 83 min & 84 min, respectively) (Fig. 2C–D). Mass spectrometry of the metabolites of **1** confirmed that the benzofuran ring was the primary site of oxidation. As predicted, replacement of the benzofuran with a fluoro-substituent reduced oxidation of the aryl ring but **2** underwent oxidative de-methylation of the tertiary amide moiety (**Table S1**). Consequently, additional medicinal chemistry-based optimization of the structure to improve its pharmacodynamic and pharmacokinetic properties will be needed before mouse efficacy studies of this scaffold can be undertaken.

### Mutations in the SAGA (Spt-Ada-Gcn5-Acetyltransferase) complex component *SGF29* cause resistance to isoxazole 1.

Our chemical-genetic observations strongly support that isoxazole **1** inhibits Acs1 as the primary mechanism of their anticryptococcal activity. To identify other potential targets or pathways that may mediate resistance, we generated isoxazole **1**-resistant mutants using a serial passaging approach. We have previously shown that aceto-acetyl CoA synthetase (Kbc1) contributes to the AcCoA pool in *C. neoformans* during mouse infection and also modulates fluconazole susceptibility (23). Since Acs1 and Kbc1 are mechanistically similar, it seemed possible that Kbc1 could play a role in either the activity of, or the resistance to, **1**. Therefore, we subjected three strains to serial passaging experiments: the H99 reference strain, the *acl1*Δ mutant and a strain lacking both *ACL1* and the aceto-acetyl CoA synthetase *KBC1* (*acl1*Δ *kbc1*Δ mutant, ref. 23).

The stains were incubated in microtiter plates containing RPMI/MOPS medium and isoxazole **1** at an initial concentration of ½ MIC for each strain. After 72hr, surviving cells were diluted 1:1000 and transferred to plates with 2-fold higher concentrations of **1** compared to the previous incubation. This transfer was repeated to a maximum concentration of 64 μg/mL which is the limit of solubility for **1** in these conditions. Approximately 90% of lineages went extinct before the 64 μg/mL concentration was reached. Random clones of the three lineages were passaged on YPD without isoxazole **1** and re-tested at 64 μg/mL of **1** to confirm stable resistance. A total of four isolates encompassing the three parental lineages were initially screened by Sanger sequencing of the *ACS1* gene; however, no *ACS1* mutations were identified in these isolates.

**Figure 4.**
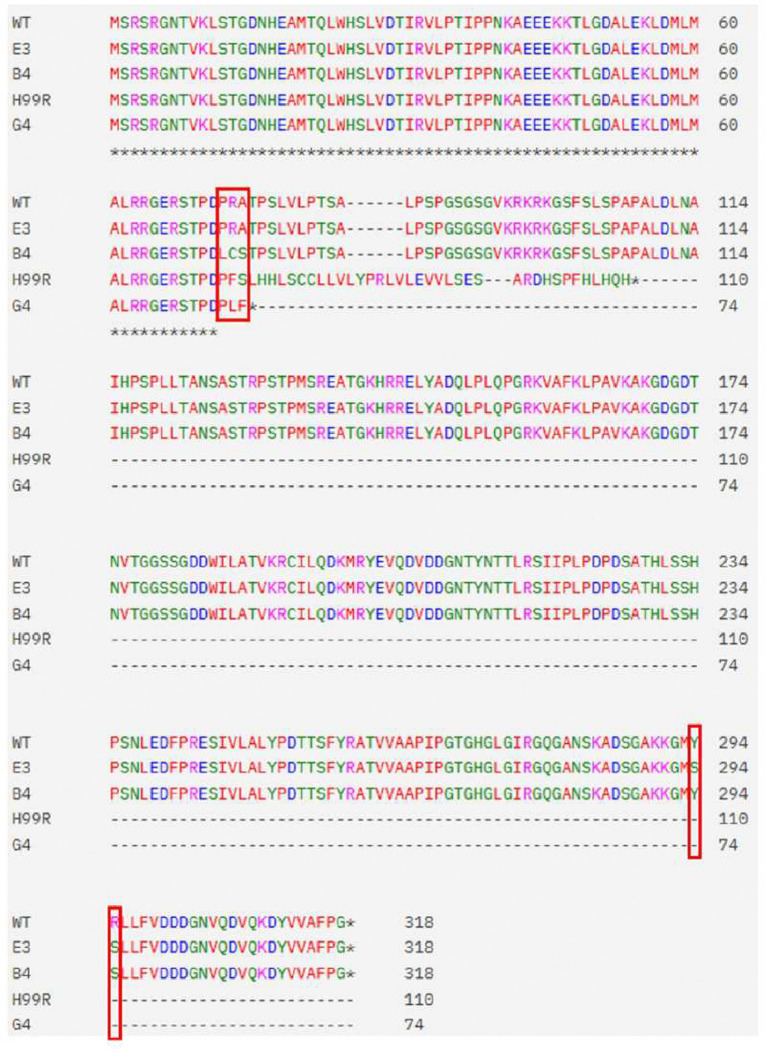
*SGF29* mutations in isoxazole 1-resistant strains. Alignment of the Sgf29 coding region for parental H99 and the isoxazole **1**-resistant isolates. Isolates H99R (derived from H99) and G4 (derived from the *acl1Δ kbc1Δ* double mutant) have insertion mutations leading to frame-shift truncation of Sgf29 at amino acid 110 and 74, respectively. Red boxes indicate the location of mutations in the full-length proteins for resistant isolates E3 and B4 (both derived from *acl1Δ* mutant). The mutations at 294 and 295 are in the Tudor domain of the protein. See Supplemental Table 2 for identify for base changes corresponding to each mutation.

Next, we performed whole genome sequencing of the parental and isoxazole **1**-resistant isolates as described in materials and methods. The sequences were mapped to the H99 reference strain and SNPs in each parental strain relative to the reference were initially identified. Consistent with the Sanger sequencing results, no SNPs were identified in *ACS1*. Each of the four isoxazole **1**-resistant strains contained a loss of function allele of the SAGA complex component *SGF29* (CNAG_06392). As shown in Fig. 4 and **Table S2**, the mutations were either INDELs or missense mutations leading to early stop codons or modification of the highly conserved N-terminal Tudor domain of Sgf29 (31, 32). No other mutations were identified in coding regions that were shared by all four isolates. Five additional proteins had non-synonymous SNPs but none were clear loss of function mutations (**Table S2**). Based on extensive studies in the model yeast *S. cerevisiae*, Sgf29 recognizes methylated histones via its Tudor domains and recruits the SAGA complex where it mediates acetylation of the histones and transcriptional activation (31) and this function has been confirmed in *C. neoformans* (32).

**Figure 5.**
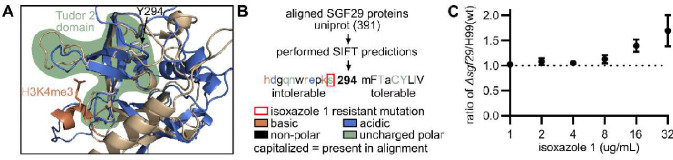
*SGF29* deletion mutant is resistant to isoxazole 1. **A.** Homology model of Sgf29 showing predicted location of *sgf29^Y294S^* mutation. **B.** Summary of SIFT analysis of *sgf29^Y294S^* mutation indicating that it is predicted to be intolerable. **C.** Competitive growth assay between mNEON-tagged H99 and *sgf29Δ* mutant at the indicated concentrations of isoxazole **1**. The ratio of the two strains was determined by flow cytometry with data represented as mean and standard deviation of three independent replicates.

Previously, the crystal structure of the human Sgf29 homolog bound to the histone H3K4me3 peptide was solved (PDB: 3ME9). We utilized AlphaFold to construct a model of *C*. *neoformans* Sgf29 (33) and aligned it to 3ME9. This revealed that multiple mutations clustered within the Tudor domain (position 292–295). Of these, Y294S mutation is predicted to be deleterious using the Sorting Intolerant From Tolerant (SIFT) tool (34) when compared to all Sgf29 amino acid sequences available in UniProt (Fig. 5 A–B). These mutations and modeling strongly suggest that loss or reduced function of SGF29 leads to isoxazole **1** resistance in C. neoformans. We, therefore, tested the susceptibility of a strain with a targeted deletion of *SGF29* and found it was also resistant to isoxazole **1** relative to its H99 parental strain (MIC >64 μg/mL). We also compared the competitive fitness of the *sgf29*Δ mutant to its H99 parental strain using a flow-cytometry-based assay (35). In that assay, a 1:1 mixture of mNEON-labeled H99 and the unlabeled *sgf29*Δ mutant was incubated with DMSO solvent or isoxazole **1** (32 μg/mL) in RPMI/MOPS medium overnight at 37°C. No difference in competitive fitness was noted in the absence of **1**, but the *sfg29*Δ mutant was significantly more fit than H99 in the presence of **1** (Fig. 5C). These data strongly support the conclusion that loss of SAGA acetyl transferase activity improves the fitness of *C. neoformans* when AcCoA synthase activity is compromised.

Interestingly, prior work showed that serial passage of the ACS inhibitor AR-12 in *S. cerevisiae* led to a resistant-isolate with a mutation in *TRA1*, another component of the SAGA complex (16). The acetyl transferase activity of the SAGA complex uses AcCoA as one of its substrates, suggesting that there may be a compensatory response linking loss of SAGA function to altered dependence of Acs1 for AcCoA homeostasis. Regardless of the specific molecular mechanism underlying the interaction between *SGF29* and *ACS1*, these data provide support for the conclusion that the antifungal activity of isoxazole **1** is due in large part to its effect on Acs1 and AcCoA homeostasis.

### Isoxazole 1&2 are highly selective inhibitors of *Cn*Acs1.

**Figure 6.**
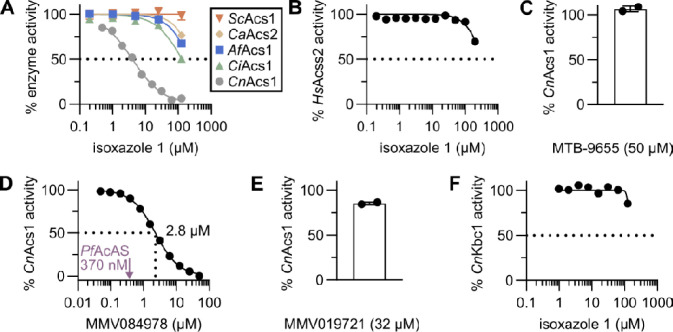
Isoxazole 1 is highly selectivity for CnAcs1 relative to other fungal ACS and human ACSS2 enzymes. **A.** The activity of isoxazole **1** against purified *Saccharomyces cerevisiae* Acs1 (ScAcs1), *Candida albicans* Acs2 (CaAcs2), *Aspergillus fumigatus* Acs1 (AfAcs1), *Coccidioides immitis* Acs1 (CiAcs1), and *Cryptococcus neoformans* Acs1 (CnAcs1). The inhibition curves are representative of three independent experiments showing similar results. **B**. Isoxazole **1** has minimal activity toward human ACSS2. **C**. Single dose experiment assessing the activity of the human ACSS2 inhibitor MTB-9655 against CnAcs1 at the maximum soluble concentration. Bar indicates mean of two independent experiments with error bars showing standard deviation. **D**. Antimalarial, *Pf*AcAS inhibitor MMV084978 inhibits CnAcs1 with IC_50_ = 2.8 μM. The IC_50_ toward *Pf*AcAS is indicated by arrow. **E**. *Pf*AcAS inhibitor MMV019721 does not inhibit CnAcs1 at the maximum soluble concentration. **F**. Isoxazole **1** does not inhibit the *C. neoformans* aceto- acetyl CoA synthetase *Cn*Kbc1.

The isoxazole *Cn*Acs1 inhibitors are structurally distinct from other fungal, human and *Plasmodium falciparum* ACS inhibitors reported in the literature (11, 17, 18, 36, 37). Therefore, we were interested in determining their spectrum of activity as ACS inhibitors. In prior work (25), we characterized the enzymology and structural basis of substrate selectivity for *Cn*Acs1 as well as multiple other fungal ACS enzymes. As part of those studies (25), His-tagged ACS enzymes from *C*. *neoformans*, *C. albicans*, *Aspergillus fumigatus*, and *Coccidioides immitis* were expressed in *E. coli* and purified by immobilized metal affinity chromatography (**IMAC, Fig. S3**). ACS activity was then measured in a coupled kinetic assay that detects pyrophosphate product release as previously reported (25). We used a 6-point 5-fold concentration series of **1** to assess initial IC_50_ values for each enzyme. As discussed above, the structures and sequences of the fungal ACS enzymes are highly similar and conserved, respectively. Therefore, we were surprised to find that isoxazole **1** was only able to inhibit *Cn*Acs1 (IC_50_ = 8.6 ± 4.4μM) (Fig. 1& 6A). Consequently, it appears that the lack of antifungal activity of **1** toward *C. albicans* and *C. glabrata* is due to its selectivity for the *Cn*Acs1 enzyme and its inability to inhibit *Ca*Acs2, the essential ACS in *C. albicans*.

To further explore the selectivity of isoxazole **1**, we expressed and purified the human ACSS2 (**Fig. S3**). Consistent with the selectivity observed amongst fungal ACSs, **1** did not inhibit ACSS2 at the limit of its solubility (Fig. 6B). This result explains the lack of in vitro toxicity for **1** toward the Hep2G cell line (Fig. 3A). Interestingly, we have previously reported that an isoxazoline inhibitor of ACSS2 has no activity toward *Cn*Acs1 (23). A second class of ACSS2 inhibitors, MTB-9655 has also become commercially available and is currently in clinical trials for cancer therapy (38). Again, MTB-9655 has no activity against *Cn*Acs1 (Fig. 6C). Finally, we also tested MMV084978 and MMV019721, two molecules that inhibit *Pf*AcAs and have anti-malarial activity (17). MMV084978 inhibited *Cn*Acs1 with an IC_50_ of 2.8 μM which is ~8-fold higher than the IC_50_ reported for *Pf*AcAs (370 nM) inhibition (Fig. 6D, ref. 17), and no IC_50_ could be determined for MMV019721 below its solubility limit while the IC_50_ reported for *Pf*AcAs is 73 nM (Fig. 6E, ref. 17). These observations further support the conclusion that different chemical classes of ACS inhibitors can show selectivity toward enzymes from different species.

Acyl CoA synthetases with different carboxylic acid specificities have similar overall biochemical and structural mechanisms (10). Consequently, we next asked if isoxazole **1** was selective for ACS when compared to a different acyl-CoA synthetase. *C. neoformans* Kbc1 converts aceto-acetate, a substrate structurally very similar to acetate, to aceto-acetyl CoA but has almost no activity in the conversion of acetate to AcCoA (13). As shown in Fig. 6F, **1** did not inhibit the acetoacetyl-CoA synthetase *Cn*Kbc1 (23). Taken together, these data indicate that isoxazole **1** displays remarkable selectivity for *Cn*Acs1, the enzyme upon which the chemical genetic screen was designed. This selectivity is observed despite the mechanistic, sequence, and structural similarities among other fungal ACS enzymes and the human acetyl CoA synthetase, ACSS2 (25).

### Isoxazole 1 is an uncompetitive inhibitor of *Cn*Acs1.

The structural biology and biochemistry of the active site of multiple ACS enzymes has been characterized primarily through analysis of bi-substrate alkyl adenine-monophosphate ester (alkyl-AMP) inhibitor-enzyme complexes (10, 25). Again, the conserved nature of the active sites would suggest that inhibitors identified for one enzyme would likely be inhibitors of others. Indeed, this homology was part of our original premise for the *C. neoformans* based synthetic lethal screen. In distinct contrast to this premise, our screen identified a highly selective class of ACS inhibitors. Furthermore, previous studies of antimalarial ACS inhibitors (17) as well as data shown in Fig. 6 suggest this selectivity is not unique to the isoxazole ACS inhibitors. To understand the potential mechanisms underlying this selectivity, we characterized the kinetics of *Cn*Acs1 inhibition by the isoxazoles in more detail.

**Figure 7.**
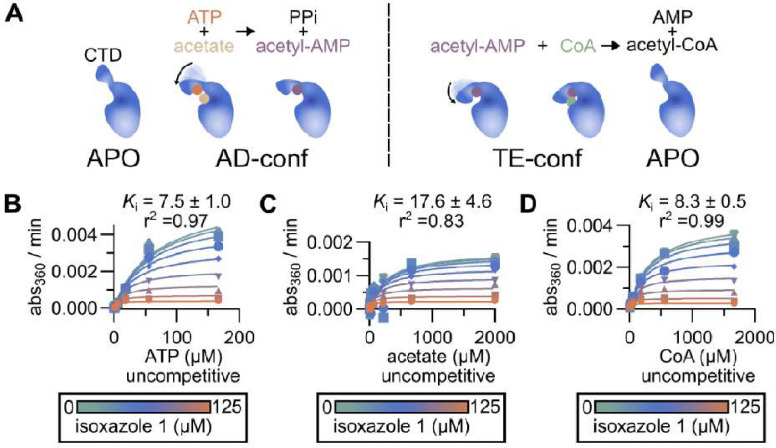
Isoxazole 1 is an uncompetitive inhibitor of CnAcs1. **A.** Schematic of the two-step reaction catalyzed by ACS and the conformational changes that occur during the reaction. APO indicates enzyme without substrate or product bound. AD indicates conformation associated with the **ad**enylation reaction that generates the Ac-AMP intermediate. TE indicates the conformation associated with the **t**hio-**e**sterification reaction of Ac-AMP with CoA to yield AcCoA. CTD indicates the **C-t**erminal **d**omain of the protein that undergoes rearrangement through the course of the reaction. **B-D**. Determination of isoxazole **1** *K*_*i*_ values for the three substrates and goodness-of-fit (*r^2^*) values for an uncompetitive model of inhibition. The color scheme indicates the color for each of the different concentrations for the reaction plots.

ACS is a multi-substrate enzyme that catalyzes the two-step conversion of acetate to AcCoA (10, 23). In the first step of the reaction, ATP condenses with acetate to generate the reactive acetyl-adenylate intermediate and pyrophosphate; in the second step, CoASH reactions with the acetyl-adenylate intermediate to generate the final product AcCoA and release AMP (Fig. 7A). The biochemical mechanism of this two-step reaction has been well-characterized and is classified as a bi-uni-uni-bi ping pong reaction (25). During this two-step biochemical reaction, the ACS enzyme undergoes a set of conformational changes as outlined in Fig. 7A (10). The apo enzyme binds ATP and acetate which leads to the AD (**ad**enylation) conformation. Next, the acyl-AMP bound form of the enzyme converts to the TE or **t**hio**e**ster conformation to allow CoASH to enter the active site.

Previously characterized ACS inhibitors, such as alkyl-AMP esters and AR-12, are ATP competitive (15, 23). Consistent with these kinetic data, alkyl-AMP esters have been shown to bind the active site and occupy the putative adenine pocket (25). Unlike these previously characterized bi-substrate inhibitors, isoxazole **1** did not exhibit a competitive mode of inhibition for any of the three substrates: acetate, CoA, or ATP (Fig. 7B–D). The isoxazole **1** data best fit an uncompetitive mode of inhibition, suggesting that it interacts with enzyme when it is bound to one or more of the substrates. To further explore the mechanism of *Cn*Acs1 inhibition by the isoxazoles, we took a structural biology approach.

### Structural analysis of isoxazole 1 bound to *Cn*Acs1.

**Figure 8.**
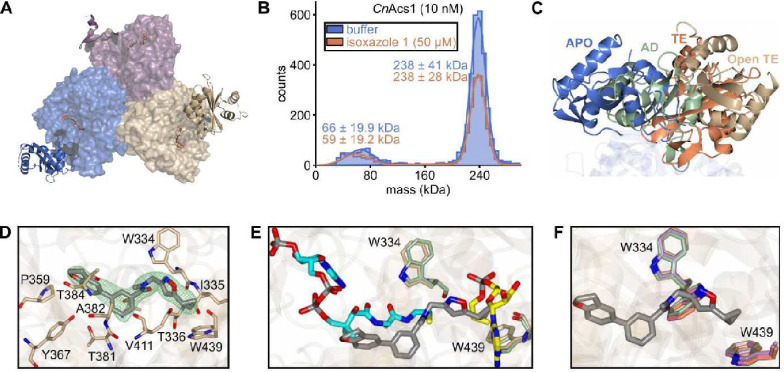
X-ray crystal structure of CnAcs1-isoxazole complex. **A**. Overall structure of the trimeric CnAcs1-isoxazole **1** complex with the C-terminal domains (CTD) shown in ribbon format. **B.** Mass photometry showing that CnAcs1 is most consistent with a trimer in solution to low concentrations and that the addition of supra-inhibitory of isoxazole **1** does not change the apparent size of the CnAcs1 protein complex. **C.** Schematic comparing “open-TE” (tan) conformation of the CTD observed in the CnAcs1-isoxazole **1** complex to the CTD conformations in the uninhibited APO (blue), AD (green), and TE (orange) forms of the protein. **D**. The region of the protein bound by isoxazole **1** (green) and its position within that pocket. **E.** Overlay of isoxazole **1** (grey) with the bound pose of Coenzyme A (turquoise) and an ethyl-AMP inhibitor (yellow) in a previously reported structure of CnAcs1 (ref. 23) showing at I interacts with both the Coenzyme A pocket and the acetyl portion of the AcAMP binding pocket. **F**. The W334 residue functions to open the CoA tunnel by rotating upon CoA binding. The position of W334 CnAcs1-isoxazole **1** (grey) complex overlaps with that observed in CnAcs1 structures with CoA bound.

To better understand the mechanism by which isoxazole **1** inhibits *Cn*Acs1, we obtained a co-crystal of it bound to *Cn*Acs1 (PDB: 9CD8, **Table 1**). The overall structure of the enzyme is similar to previous structures obtained for *Cn*Acs1 such as the complex with the propyl-AMP ester (PDB 5K85). Specifically, the *Cn*Acs1-isoxazole **1** co-crystal structure showed an overall RMSD of 1.42 Å between Ca atoms (523 residues). Consistent with previous fungal ACS structures (25, 39), the *Cn*Acs1-isoxazole **1** co-structure is a trimer (Fig. 8A). This trimeric structure was also observed using single molecule solution-based mass photometry (Fig. 8B), suggesting that the trimeric conformation is unlikely to be an artifact of the crystallization process.

An important structural characteristic of ANL-family adenylating enzymes is that the C-terminal domain (CTD) undergoes large conformational changes between the two biochemical steps of the overall conversion of acetate to AcCoA (10, 25). In the ACS enzyme without bound substrates (Apo form), the CTD adopts a unique conformation (Fig. 7A). The first reaction is the adenylation step in which ATP reacts with acetate to generate an acetyl-AMP intermediate with release of pyrophosphate. This step of the reaction leads to the **ad**enylation (AD) conformation. The acetyl-AMP bound enzyme then undergoes a conformational change in which the CTD rotates before the reaction between the acetyl-AMP and CoA; accordingly, this conformation is referred to as the **t**hio-**e**sterification or TE conformation. In previous work, we obtained crystal structures for CnAcs1 in each of these conformations with either substrates, intermediates, or inhibitors bound (23). The CTD in these structures is frequently observed in multiple conformations within the asymmetric units.

In the *Cn*Acs1-isoxazole **1** structure, the CTD is observed in two conformations (the CTD is disordered in one chain of the asymmetric unit). First, the CTD adopts the Apo conformation indicative of an enzyme without bound substrates while the second conformation is structurally distinct from the canonical conformations observed previously (Fig. 8A, C). This new conformation is most closely related to the TE conformation; the transition between the AD and TE conformations occurs through a hinge region upon which the CTD rotates. In the *Cn*Acs1-isoxazole **1** structure, the CTD is rotated much further relative to the hinge region than in other structures as shown in Fig. 8C. In the TE conformation, the CTD forms the interior of the CoA binding pocket. The “over-rotation” caused by isoxazole **1** binding, prevents the formation of an intact, CoA-binding tunnel and opens the region to solvent. Accordingly, we refer to this new CTD conformation as the TE-open. Isoxazole **1**-bound *Cn*Acs1 enzyme, therefore, does not recapitulate the secondary and tertiary structure *Cn*Asc1 conformations associated with the binding of substrates and substrate mimics. These observations are consistent with the fact that **1** is not competitive with ACS substrates and displays an uncompetitive mode of inhibition.

Consistent with its effect on the formation of the CTD-dependent CoA binding pocket, isoxazole **1** binds in the region that accommodates CoA with the biphenyl moiety overlapping with the CoA pantothenate chain (Fig. 8D–E). The amide and isoxazole substituents of **1** extend from the CoA binding pocket into the region occupied by the acetyl group of the acetyl-AMP intermediate or the propyl group of propyl-AMP ester inhibitors (Fig. 8E). The size of the CoA tunnel nearest to the acetyl-AMP binding pocket is modulated by a key tryptophan residue (W334, 23). In the absence of CoA, W334 rotates to allow hydrogen bonding with substrates or intermediates within the ATP or acetyl-AMP pocket. Upon CoA binding, W334 rotates to open up the CoA tunnel, presumably to allow binding of CoA and position it for the TE reaction. In the *Cn*Acs1-isoxazole **1** co-structure, W334 adopts an orientation similar to that of the CoA-bound enzyme, further supporting a model in which the biphenyl portion of the molecule functions as CoA/pantothenate mimic (Fig. 8F).

Like many ACSs, *Cn*Acs1 is exquisitely specific for acetate with little to no activity toward larger alkyl carboxylic acids such as propionate and butyrate (23, 25). Based on structural and genetic data studies of ACSs from multiple species, the indole ring of a highly conserved tryptophan (W439 in *Cn*Acs1) limits the size of the acetyl-AMP pocket and is a key determinant of substrate specificity and inhibitor binding (25). The cyclopropyl group of isoxazole **1** is positioned within the acetyl-AMP pocket in a manner similar to that observed in our previously reported structures of *Cn*Acs1 bound to linear chain (25), alkyl-AMP ester bi-substrate inhibitors.

Recently, we used an expanded series of alkyl-AMP ester bi-substrate inhibitors to probe the steric properties of alkyl groups that could be accommodated by the tryptophan wall in *Cn*Acs1 (40). The most potent alkyl-AMP-based inhibitor of *Cn*Acs1 is ethyl-AMP (8 μM) while increasing the length of the alkyl chain to propyl and butyl decreases potency 3- and >50 fold, respectively; importantly, these trends parallel alkyl carboxylate substrate specificity (23, 40). Interestingly, the IC_50_ of the cyclopropyl-AMP ester (9 μM) is essentially identical to that of the ethyl-AMP inhibitor indicating that the pocket is able to bind this alkyl group. Furthermore, we obtained an X-ray crystal structure of *Cn*Acs1 bound to the cyclopropyl-AMP ester and observed that the cyclopropyl moiety of the inhibitor and the W439 residues overlap almost exactly (PDB: 8G0T, **Table 1, Fig. S5**). Thus, the cyclopropyl-isoxazole portion of **1** binds the alkyl region of the acetyl-AMP ester pocket (**Fig. S5**).

Taken together, these structural data indicate that isoxazole **1** functions as a novel type of bi-substrate ACS inhibitor in which the cyclopropyl-isoxazole portion of the molecule interacts with the alkyl group binding portion of the acetyl-AMP pocket while the biphenyl amide extends into the CoA tunnel and disrupts its complete formation by altering the position of the CTD. In addition, the structural features of this mode of inhibition are consistent with the biochemical data indicating that isoxazole **1** is an uncompetitive inhibitor of *Cn*Acs1.

### Molecular dynamics and structure-activity relationships identify molecular determinants for the interaction of *Cn*Acs1 and isoxazole 1.

**Figure 9.**
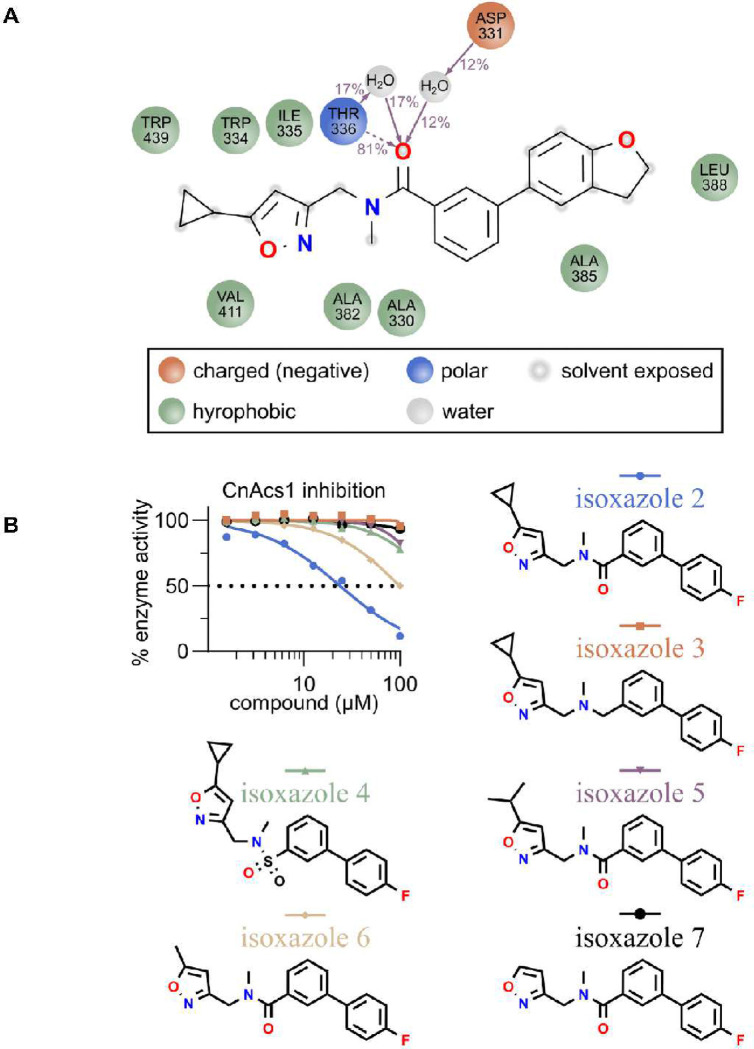
Molecular dynamics and structure-activity relationship data provide insights into interactions contributing to isoxazole 1/2-CnAcs1 binding. **A.** Schematic represents details of the isoxazole **1** interactions with each protein residue. Interactions that occur more than 11% of the simulation time in the selected trajectory (0.00 through 1000.00 nsec), are shown. Dotted arrows indicate direct interactions between protein and ligand. Solid arrows represent interactions mediated by a water molecule. The majority of residues predicted to contribute to binding participate in hydrophobic interactions (green). A key H-bonding interaction between Thr336 and the amide carbonyl of isoxazole **1** was identified in 81% of simulations. **B**. IC_50_ curves for isoxazoles **2–7** indicating that the amide carbonyl (isoxazole **3/4**) and isoxazole cyclopropyl moiety (isoxazoles **5–7**) are key drivers of the CnAcs1 potency of isoxazoles **1** and **2**.

As discussed above, the CTD does not form a complete CoA tunnel in the *Cn*Acs1-isoxazole **1** co-crystal structure. The portion of isoxazole **1** that overlaps with the pantothenate portion of CoA is exposed to solvent in the partially formed CoA tunnel. As a result, it appears that the binding of isoxazole **1** within that partially-formed tunnel is dependent upon mainly hydrophobic interactions. To further characterize the interactions that might contribute to the binding of isoxazole **1** to *Cn*Acs1, we performed molecular dynamics simulations over a 1000 nanosecond time scale using Schrödinger software as described in the materials and methods. Fig. 9A summarizes the residues of *Cn*Acs1 that interact with **1** and *Cn*Acs1 in 10% or more of the simulations. Consistent with the previous discussion regarding the importance of Trp334 interactions, this hydrophobic interaction is prominent within the simulations and a number of other residues including Trp439 appear to contribute to binding through hydrophobic interactions. The exception to this pattern is a predicted direct H-bond between Thr336 and the amide carbonyl of isoxazole **1**. The amide carbonyl and Thr336 also appear to participate in H_2_O-bridges that may also involve Asp331. These molecular dynamics simulations and the co-crystal structure suggest that the amide carbonyl and the cyclopropyl substituent are likely to be key determinants of the potency of isoxazole **1** toward *Cn*Acs1.

To test this hypothesis, we synthesized a set of molecules derived from isoxazole **2**, the more metabolically stable derivative of **1**. First, we tested the necessity of the amide carbonyl group for inhibition by synthesizing a derivative of **2** which has a methylene group bridging the biphenyl with the nitrogen (compound **3**). Consistent with our hypothesis, **3** had no activity against *Cn*Acs1 (Fig. 9B). We also synthesized the corresponding sulfonamide derivative **4** but it too was unable to inhibit *Cn*Acs1. Lastly, we varied the nature of the isoxazole alkyl groups to determine the range of substituents that would be tolerated at this position. As indicated in Fig. 9B, the cyclopropyl group is critical for the potency of these isoxazole. The very small structural change from cyclopropyl to isopropyl (5) eliminated inhibition entirely. Similarly, the methyl (6) and hydrogen substituted isoxazoles (7) were also inactive. In the alkyl-AMP ester series, the IC_50_ of the isopropyl-AMP ester was equivalent to that of the cyclopropyl-AMP ester. Therefore, despite the cyclopropyl substituent occupying a very similar position within the acetyl-AMP ester binding pocket as other alkyl-AMP groups, it appears to be a privileged substituent in the isoxazole series of *Cn*Acs1 inhibitors.

## Discussion

ACS has emerged as a drug target for the treatment of a variety of human diseases and infections including cancer, fatty liver disease, malaria, and mycoses (13–18). One of the reasons that ACS is an attractive target for the treatment of human disease is that under normal physiological conditions ACSS2 and its substrate, acetate, are minor contributors to the overall pool of AcCoA in human or mammalian cells (13, 14). Instead, glucose is the major carbon source from which AcCoA is derived, through the TCA cycle and the enzyme ATP-citrate lyase; accordingly, deletion of ACSS2 is possible in mice while deletion of ATP-citrate lyase is not. However, ACSS2 has been shown to be involved in the generation of AcCoA in specific physiological circumstance in mammals (41, 42), indicating that loss of its function may have more subtle effects on cellular function. Consequently, in the context of targeting ACS as an approach to treating infections, the selective inhibition of microbial, parasitic or fungal ACS enzymes relative to host ACSS2 would be likely to reduce potential adverse effects of those drugs.

With these considerations in mind, the first general conclusion from this work is that highly selective inhibition of ACS enzymes is chemically and biochemically feasible. To date, three other ACS inhibitors identified by phenotypic or target-based high throughput screening have been reported (11, 17, 36). Above, we described the identification of isoxazole **1** through a chemical-genetic, phenotypic screen using *C. neoformans*. Somewhat surprisingly, **1** has no activity against other fungal ACS enzymes and is inactive toward the human enzyme ACSS2. The isoxazoline ACSS2 inhibitor VY-3–249 was identified by an in vitro screen directly against the ACSS2 enzyme (11) and also has no activity against any of the fungal ACS enzymes examined to date (25). Interestingly, a more potent analog, VY-3–135, is selective for ACSS2 over the related human enzymes ACSS3 (36). Similarly, an ACSS2 inhibitor currently in clinical trials for a cancer indication, MTB-9655, also has no activity against *Cn*Acs1 (Fig. 5C). The antimalarial ACS inhibitor MMV0894978 was identified in a phenotypic screen for growth inhibition (17). MMV0894978 inhibits *Pf*AcAs, *Cn*Acs1 and ACSS2 but with IC_50_ values that are 8- and 50-fold higher toward *Cn*Acs1 and ACSS2, respectively. This selectivity is despite high levels of sequence conservation among the ACS enzymes (**Fig. S4**), particularly with respect to residues in the region of the active site. Taken together, these data and literature strongly support the conclusion that ACS enzymes from a variety of species can be selectively inhibited.

An understanding of the selectivity displayed by these chemically distinct ACS inhibitors has been hampered by a lack of X-ray crystal structures for inhibitor-enzyme co-complexes and biochemical characterization of the mechanism of inhibition for the human inhibitors. For example, no experimental structural data for ACSS2 is available and the mechanism of those inhibitors has necessarily been inferred from homology modeling and docking studies (17, 36). For the antimalarial ACS inhibitor, biochemical data indicate that MMV0894978 is uncompetitive or shows mixed inhibition kinetics with respect to all three substrates while a structurally distinct *Pf*AcAS inhibitor MMV019721 (2-fold less active than MMV0894978 toward *Cn*Acs1) was competitive with CoA (17). Mutations conferring resistance to the *Pf*AcAS inhibitors mapped to the CoA binding region of the enzyme based on a homology built from a *Cn*Acs1 structure (17). The CoA pocket of *Pf*AcAS has also been shown to be vulnerable to inhibition by CoA anti-metabolite forming pantothenamides (18).

Like the MMV inhibitors (17), isoxazole **1** does not show simple substrate-competitive inhibition kinetics but rather appears to be an uncompetitive inhibitor of *Cn*Acs1. The co-crystal structure of isoxazole **1** with *Cn*Acs1 shows that **1** engages the CoA pocket and, therefore, is likely to share mechanistic similarities to the MMV inhibitors. Based on the selectivity of the MMV- and isoxazole-ACS inhibitors for their respective targets, these data suggest that the CoA binding site may be a region of the enzyme amenable to the further development of ACS inhibitors with high specificity. It is also tempting to speculate that the *Cn*Acs1 inhibition of MMV-inhibitors may be due to their targeting the same region of the ACS active site.

The structure of *Cn*Acs1-isoxazole **1** complex also reveals that isoxazole **1** represents a novel type of bi-substrate inhibitor of the ANL-family adenylating enzymes. As discussed above, the inhibition of ANL family enzymes with molecules that mimic the acyl-AMP intermediate (i.e., bi-substrate) has proven to be very effective (23, 40). For example, acyl-AMP bi-substrate molecular mimics have been developed as inhibitors of bacterial siderophore (43), biotin (44), and vitamin K biosynthesis (45). These acyl-AMP isosteres interact with the adenosine and acyl binding regions of the adenylating enzymes. In contrast, isoxazole **1** interacts with the CoA and acyl binding regions of the active site. Accordingly, it seems more accurate to consider isoxazole **1** as a mimic of the reaction product AcCoA. However, if the isoxazoles were a simple or general AcCoA mimic then it further seems likely that they would interfere with a wide range of AcCoA-related processes and thus cause significant toxicity.

Since the isoxazoles do not cause general toxicity to eukaryotic cells and are exquisitely specific for the *Cn*Acs1 enzyme, their ability to mimic AcCoA appears to be dependent upon the presence of the *Cn*Acs1 enzyme and its interactions with the CoA/acyl binding pocket. Supporting this qualitative assertion, the minimized unbound conformation of isoxazole **1** is 4.8 kcal/mol more stable than the conformation observed in the co-crystal with CnAcs1 as determined by molecular mechanics calculations (MMGBSA, Schrödinger, Fig. S6). Therefore, isoxazole **1** undergoes a significant conformational re-organization upon interaction with the CoA binding pocket of *Cn*Acs1. This re-organization is somewhat reminiscent of an “induced-fit”-like ligand-enzyme binding mechanism and we propose that this may contribute to the high selectivity for this specific ACS enzyme. Importantly, this analysis suggests that synthesis of structural analogs of **1** with predicted conformations that match the bound conformation may lead inhibitors with improved potency. For example, the Myers group has recently reported the successful application of such a strategy to the synthesis of iboxamycin analogs with improved antibiotic activity through pre-organizing the molecule into its bound conformation (46).

In addition to displaying remarkable selectivity, the isoxazole *Cn*Acs1 inhibitors also provide proof-of-principle for inhibition of fungal ACS as an antifungal strategy even in species that can generate AcCoA through ATP-citrate lyases. Three considerations support this conclusion for the potential of ACS inhibitors for the treatment of Cryptococcus, in particular. First, chemical inhibition of *Cn*Acs1 inhibits growth under host-like conditions to a greater extent than deletion of the gene, suggesting that cells cannot adjust metabolic flux quickly enough to compensate for loss a pathway contributing to the total cellular AcCoA pool. Second, prior results from our lab have shown that the role of *Cn*Acs1 in carbon metabolism is even more pronounced during infection (23), supporting the possibility that the efficacy *Cn*Acs1 inhibitors is likely to be increased in vivo relative to in vitro.

Third, the AcCoA pool is critical for the synthesis of ergosterol, the key sterol of fungal plasma membranes and a target for two of the three antifungal drugs currently used to treat patients. Consistent with this role, the combination of ACS inhibitors with fluconazole is synergistic in vitro and *C. neoformans acs1*Δ mutant is hypersensitive to fluconazole in vivo (23). As a single therapy fluconazole is inferior to combination therapy for the treatment of cryptococcal meningitis (47). Therefore, there has been strong interest in developing new approaches to fluconazole-based combination therapy (28) and ACS inhibitors appear to be a strong candidate in this regard.

In summary, our discovery of the isoxazole ACS inhibitors and the structural and biochemical characterization of their mechanism of inhibition provides novel provides general insights that should be useful to the design of ACS inhibitors for both infectious disease indications as well the treatment of human cancers and metabolic diseases.

## Materials and methods

### Strains, media, and growth conditions.

Lab strains, clinical isolates, and genetically modified strains of *C*. *neoformans* or *C*. *albicans* were maintained in glycerol stocks that were stored at −80°C and recovered on yeast peptone dextrose (YPD, 1% w/v yeast extract, 2% w/v peptone, 2% w/v dextrose, and 2% w/v agar). Strains were not maintained on YPD longer than 14 days to be kept fresh for each experiment performed. Overnight cultures grown into log phase shaking at 200 rpm in liquid YPD were used for each experiment. In cases where experimental procedures did not utilize YPD media the appropriate media recipes are described in the associated methods sections. Clinical strains 111.00 and 103.98 were kindly provided by John Perfect at Duke University. Our standard lab strain of *C*. *neoformans* is of the H99-stud lineage and the standard lab strain of *C*. *albicans* used in these studies is SC5314. The *C*. *neoformans acl1Δ* deletion mutant was generated and validated in previous work (23). The *C*. *neoformans sgf29Δ* deletion mutant was generated as part of the Madhani knockout collection and obtained from the Fungal Genetics Stock Center (FGSC).

### Compound Library.

We obtained and screened 55,264 compounds from the ChemBridge DiverSET library designed by ChemBridge. The library was initially provided in 10 mM DMSO stocks and then diluted to 1.25 mM in 50% DMSO for screening purposes. All compounds were stored at −80°C in 384-well plates with sealing foil. Each plate was thawed completely in a desiccating chamber before each use and minimally maintained at room temperature for the duration of assay assembly. For the initial hit validation screen, primary hits were manually picked from the original compound library while secondary screening assays were performed with compounds that were re-ordered directly from ChemBridge.

### Primary, validation, and secondary screens.

Compounds were screened against the ACS-dependent *C*. *neoformans acl1Δ* deletion mutant in a high throughput-based growth assay. On Day 0, the *acl1*Δ mutant was inoculated into 50 mL of YPD where a 1:10 dilution and 1:25 dilution culture was also created and grown shaking at 200 rpm at 30°C to early log phase overnight. The culture was adjusted to a density of 5.33 × 10^5^ cells/mL and 15 μL was added to a 384-well plate containing 10 μl of YPD and 0.5 μl of compound (Nimbus, MicroLab). The final concentrations in each well were 8000 cells, 25 μM compound, and 1% DMSO in YPD. The were incubated at 30°C for 48 hours before the optical density (OD_600_) for each plate was determined using a plate reader (SpectraMax i3X, Molecular Devices). Hits were verified using cherry-picked samples under identical conditions as the primary screen. Validated hits were reordered and counter-screened in a secondary assay where final concentrations of assay components were the same but scaled to 100 μl volume in a 96-well plate format and read as before.

### Chemical synthesis.

The synthetic methods and molecule characterization data are provided in the supplementary methods section.

### Antifungal susceptibility assays.

MICs were performed using a slightly modified Clinical and Laboratory Standards Institute CLSI standard methods (26). Overnight cultures were washed in sterile phosphate-buffered saline (PBS) brought up into either YPD, YNB-acetate, or RPMI supplemented with 165 mM MOPS pH 7.0 such that 1 × 10^3^ cells would be delivered into each well with a final volume of 200 μl in a 96-well plate. Each tested drug was added such that the final DMSO concentration did not exceed 1.28% and the maximum drug concentration tested was at least four-fold higher than the reported MIC or the limit of solubility. Plates were incubated at 37°C for 72 hours for *C*. *neoformans* and 24 hours for *C*. *albicans*, unless otherwise stated. Each assay was performed in a minimum of technical duplicates with a minimum of two independent experimental replicates. Fractional inhibitory concentration assays were performed under similar conditions to MICs but set up with a standard checkerboard dilution for each paired (16).

### In vitro toxicity assay.

HepG2 (ATCC, HB-8065) cells were maintained in Dulbecco’s modified Eagle medium (DMEM; Gibco, Cat #11965–092) supplemented with 10% fetal bovine serum (FBS) and 1% penicillin/streptomycin. Cells were cultured at 37°C in a humidified atmosphere with 5% CO_2_. For experiments, cells were seeded into 96-well plates at a density of 1.25 × 10^4^ cells per well and incubated overnight under the same culture conditions. The following day, the medium was replaced with fresh medium containing a two-fold dilution series of the test drug, with an equal concentration of DMSO in all wells. After 24 hours of incubation, the supernatant was collected to quantify lactate dehydrogenase (LDH) release using the CyQuant LDH assay kit (Invitrogen, Cat #C20300), following the manufacturer’s protocol. LDH levels were normalized to the maximum lysis control, achieved by treating cells with Triton X-100.

### In vitro characterization of microsomal stability and metabolism.

Isoxazoles **1** and **2** (10mM in DMSO) were incubated at 37°C with 0.5 mg/ml Mouse microsomes (Lot 2110246) fraction and Phase I (NADPH Regenerating System) cofactors for 0–120 min at a final concentration of 10 μM. Prior to adding compounds, microsomes were preincubated with 500uM 1-ABT (Lot 247772) (final concentration, in DMSO) for 30 minutes at room temperature. Reactions were quenched with 0.5 mL (1:1) of methanol containing formic acid 0.2%, 4mM ammonium acetate, and 100 ng/ml n-benzylbenzamide IS (0.1%FA, 2mM NH4 Acetate, and 50 ng/mL final concentration). Samples were vortexed for 30 seconds, incubated at RT for 10 min and spun for 5 min at 2200 rpm in a table-top centrifuge. Supernatants (~0.9 mL) were then transferred to an Eppendorf tube and spun in a 4°C microfuge for 5 minutes at 13200 rpm. Supernatant (800 μL) was transferred eluted through a 0.2 micron PVDF syringe filter into a LCMS vial with insert and analyzed by LC-TOF/MS. After sample acquisition, data was transferred to processing computer and loaded into MetabolitePilot for metabolite ID. Individual samples were screened individually for potential metabolites. Peaks filtering included 1) Confidence score above 45%, 2) Charge state of +1 and 1) mass accuracy within 12ppm of calculated formula. Resulting peaks were visually reviewed to ensure no background peaks were selected. All remaining peaks were selected for reporting. Results were filtered to only show peaks present in 3 or more samples. Reported metabolites were those that were present in 3 or more samples or were present in only one sample but part of a reported multi-transformation result. Peak areas were summed (across timepoints, and across descreet peaks of same transformation type), and metabolites comprising greater than 5% of total peak were investigated. The method described in McNaney, et al (47) is used with modification for determination of metabolic stability half-life by substrate depletion. A “% remaining” value was used to assess metabolic stability of a compound over time. The LC-MS/MS peak area of the incubated sample at each time point was divided by the LC-MS/MS peak area of the time 0 (T0) sample and multiplied by 100. The natural Log (ln) of the % remaining of compound was then plotted versus time (in min) and a linear regression curve plotted going through y-intercept at ln(100). If the metabolism of a compound failed to show linear kinetics at later time point, those time points were excluded. The half-life T12 was calculated as T12=0.693/slope. Intrinsic Clearance (${Cl}_{int}^{\prime }$) is calculated as described by Obach (48) using the well-stirred model:

CLint′=0.693invitroT12×mlincubationmgmicrosomes×mgmicrosomesgliver×gliverkgb.w.

### Whole genome sequencing of resistant mutants.

Isoxazole-resistant strains and their parents were grown overnight in 25 mL YPD. Pelleted cultures were transferred to a 2 mL screw cap tube with PBS, pelleted, and supernatant removed. Samples were lyophilized overnight and 0.5 mm diameter glass beads (BioSpec Products) were added the following day. Freeze dried material was subject to bead beating for 1 minute or until cell pellets was broken into a fine powder followed by the addition of 1 mL of CTAB extraction buffer [100 mM Tris-HCl pH 7.5, 700 mM NaCl, 10 mM EDTA, 1% cetyltrimethylammonium bromide (CTAB), 1% β-mercaptoethanol (14 M)]. Samples were incubated at 65° C for 30 minutes followed by the addition of 1mL Chloroform and gentle mixing. Samples were centrifuged for 10 minutes at 4,000 rpm where the aqueous phase was transferred to a clean tube for a second round of chloroform extraction. Final aqueous phase was transferred to a clean tube and followed by standard ethanol precipitation. Final samples were resuspended in nuclease-free water and sent to SeqCoast for further analysis. Received samples were prepared for whole genome sequencing using an Illumina DNA Prep tagmentation kit and unique dual indexes. Sequencing was performed on the Illumina NextSeq2000 platform using a 300-cycle flow cell kit to produce 2×150bp paired reads. 1–2% PhiX control was spiked into the run to support optimal base calling. Read demultiplexing, read trimming, and run analytics were performed using DRAGEN v3.10.12, an on-board analysis software on the NextSeq2000. We include fastqc metrics as a best practice and for examination in the case of unexpected outputs. Reads were aligned to the *C. neoformans* H99 genome (FungiDB version 52) using bowtie2 followed by variant calling via samtools and vcftools with a minimum read depth = 10 and quality PHRED score = 37. Variants identified in each parental strain were filtered out of each respective isoxazole resistance strain.

### Flow cytometry competition assay of isoxazole 1-treated H99 and *sgf29*Δ mutant.

Following a previously published protocol (35), overnight cultures of the H99 reference strain, H99-mNeonGreen expressing strain, and the *sgf29Δ* deletion strain were grown in standard YPD media. Tested strains were washed with PBS, resuspended in RPMI-MOPS pH 7.0 and mixed 1:1 with the H99-mNeonGreen expressing strain such that 1 × 10^4^ cells/mL per strain were delivered with a final volume of 100μl per well in a flat bottom 96-well plate. A dilution series of isoxazole 1 was delivered such that the DMSO concentration remained constant and did not exceed 1.25%. Co-cultures were grown overnight at 37 °C for 24 h in ambient air before analyzing on an Attune NxT Flow Cytometer with CytKick autosampler and Attune Cytometric software as previously described.

### Recombinant enzyme expression and purification.

Each recombinantly expressed and purified enzyme used in this study has been previously described. Briefly, expression plasmids were transformed into the *Escherichia coli* strain BL21 with appropriate antibiotic selection. Resistant colonies were used to start an overnight culture in standard LB broth with continued antibiotic selection shaking at 200 rpm and 37°C. The following morning LB broth supplemented with 50 mM glucose and fresh antibiotic was inoculated with 1:1000 dilution of each respective overnight culture and allowed to grow shaking at 200 rpm and 37°C until mid-log phase (OD_600_ 0.5–0.8) then induced with 1 mM isopropyl-β-D thiogalactopyranoside (IPTG) for 2 hours. Pelleted cells were lysed and protein was purified via immobilized metal affinity chromatography (IMAC) as previously described (25). All proteins were dialyzed and stored at −80° C in elution buffer.

### Enzymological characterization of ACS inhibitors.

Enzyme activity was detected using our previously described (25) absorption based coupled kinetic assay as modified from the EnzChek Pyrophosphate Assay Kit (Thermo). Substrate and coupling reagents were either prepared fresh or thawed from small aliquots stored at −80°C for a maximum of two freeze-thaw cycles. All tested drug concentrations were diluted from stock such that DMSO concentrations did not exceed 5%. All reagents including compound and minus the start reagent were mixed and aliquoted at room temperature followed by a 15-minute incubation at 37°C. The start reagent either acetate or acetoacetate in the case of *Cn*Kbc1 was then added and the reaction was followed continuously at 37°C in a SpectraMax i3X Multi-Mode plate reader (Molecular Devices) at absorbance 360 nm. Single point inhibition studies were performed with 50 μM compound and percent inhibition was normalized to a DMSO control. Dose response curves were generated using a minimum 10-point two-fold drug dilution series using concentrations indicated for each compound tested along with a DMSO control. Inhibition constants (*K*_i_)’s were determined by varying one substrate while holding the other substrate pairs in excess across a dilution series of compound 1. Substrates were supplied in excess for the respective *K*_i_ determination of compound 1 such that, ATP = 2.5 mM, CoA = 1 mM, and acetate = 0.5mM. The 50% inhibitory concentrations (IC_50_)’s and *K*_i_’s were calculated using the non-linear regression analysis software, Prism (GraphPad). All inhibition studies were performed with a minimum of two experimental duplicates and always with a positive control inhibitor, ethyl-AMP at 50 μM.

### Crystallization conditions and procedures.

Purified *Cn*Acs1 was concentrated to 10 mg/mL in 20 mM NaCl, 20 mM Tris pH 8.5, 1 mM TCEP for crystallization screening. All crystallization experiments were setup using an NT8 drop-setting robot (Formulatrix Inc.) and UVXPO MRC (Molecular Dimensions) sitting drop vapor diffusion plates at 18 °C. 100 nL of protein and 100 nL crystallization solution were dispensed and equilibrated against 50 uL of the latter. **Isoxazole 1-CnAcs1**: The complex with isoxazole **1** was prepared by adding the inhibitor, from 100 mM stock in DMSO, to an aliquot of the protein to a final concentration of 2 mM and incubating for 30 minutes on ice. Crystals were obtained in 1–2 days from the Index HT screen (Hampton Research) condition F2 (0.2 M Ammonium sulfate, 0.1 M HEPES pH 7.5, 25% (w/v) PEG 3,350). Samples were transferred to a fresh drop composed of 80% crystallization solution and 20% (v/v) PEG 200 and stored in liquid nitrogen. **Cyclopropyl-AMP-CnAcs1**: 12.5% 8K, 200 mM NaCl, 100 mM K/Na phosphate pH 6.2. 1 mM ligand was added to the protein prior to crystallization. Samples were transferred to 25% (v/v) PEG 200 + 75% crystallant for cryoprotection. X-ray diffraction data were collected at the National Synchrotron Light Source II (NSLS-II) beamline 19-ID (NYX).

### Structure solution and refinement.

Intensities were integrated using XDS (50) via Autoproc (51) and the Laue class analysis and data scaling were performed with Aimless (52). Structure solution was conducted by molecular replacement with Phaser (53) using a previously determined structure of *Cn*Acs1 (PDB 8EPS) as the search model. Structure refinement and manual model building were conducted with Phenix (54) and Coot (55), respectively. Structure validation was conducted with Molprobity (56) and figures were prepared using the CCP4MG package (57).

### Molecular dynamics calculations.

The docked ligand-receptor complex was subjected to molecular dynamics (MD) simulation using the Desmond module of Schrodinger software (Schrodinger, LLC, New York, NY, 2024–1) with OPLS 4 force field (58). First, the molecular system of the ligand-receptor complex was built with water molecules in a cubic box via the simple point charge (SPC) method; which was later followed by ions neutralization by the addition of sodium, to balance the net charge of the solvated system. MD simulation was performed for 1000 ns using the Isothermal-isobaric (NPT) ensemble class, where temperature and pressure were reserved at 300 K and 1.01325 bar pressure employing Nose-Hoover temperature coupling and isotropic scaling. Equilibration of the systems and MD simulations were carried out using the default protocol provided in Desmond (59).

### Molecular docking calculations.

The chemical structure of the isoxazole **1** was drawn using ChemDraw Ultra 12.0 followed by 3D optimization using Marvin Suite 5.5.1.0. Molecular docking of the ligand was performed using Maestro 13.9.138 in the Schrodinger software (Schrodinger, LLC, New York, NY, 2024–1). Ligand was prepared by the LigPrep tool, using an OPLS 4 force field. Ligand was optimized by generating desalt states, ionization, and tautomerization variations and generating a physiological pH value of 7.0 ± 2.0 using Epik structure. The protein structure (CnACS) was run through the Protein Preparation in Maestro to ensure the protein was suitable for modeling calculations (60). For that, bond orders were assigned, missing hydrogens/side chains were added, corresponding to pH 7.0, and waters were deleted located beyond 6Å from receptor hetero groups. OPLS 4 force field was used to minimize protein energy. Then, minimized protein was used to generate a grid box at the centroid of the active site for docking and was created by picking the isoxazole **1** ligand structure. For ligand docking, a receptor grid generated .zip file and ligprep file were used. The glide module was used with the standard setting of Extra Precision (XP) mode (61) for the scoring process and keeping ligands flexible and receptors rigid except at the active site.

### SRA accession numbers.

Whole genome sequencing data have been submitted to the National Center for Biotechnology Information (NCBI) Sequence Read Archive (SRA) under PRJNA1190938.

### PDB codes for structures.

Coordinates and structure factors for *Cn*Acs1 inhibitor complexes have been deposited to the Worldwide Protein Databank (wwPDB) with the accession codes 9CD8 (isoxazole **1**) and 8G0T (cyclopropyl-AMP).
